# Risk factors and mortality in infants who received bag and mask ventilation at birth: a secondary analysis from the global network maternal newborn health registry

**DOI:** 10.1038/s41372-026-02732-8

**Published:** 2026-06-01

**Authors:** Zubair H. Aghai, Avinash Kavi, Vanessa R. Thorsten, Manjunath S. Somannavar, Ramachandra Bhat, Elizabeth M. McClure, Denise C. Babineau, Archana B. Patel, Sarah Saleem, Sangappa M. Dhaded, Umesh Y. Ramdurg, Blair J. Wylie, Manolo Mazariegos, Antoinette K. Tshefu, Waldemar A. Carlo, Nancy F. Krebs, William A. Petri, Patricia L. Hibberd, Edwin J. Asturias, Sherri L. Bucher, Jackie K. Patterson, Melissa S. Bauserman, Fabian Esamai, Robert L. Goldenberg, Rupsa C. Boelig, Shivaprasad S. Goudar, Richard J. Derman

**Affiliations:** 1 Nemours Children’s Health System, Wilmington, DE USA; 2 Jawaharlal Nehru Medical College, KLE Academy of Higher Education and Research, Belagavi, India; 3 RTI International, Research Triangle Park, NC USA; 4 Lata Medical Research Foundation, Nagpur, India; 5 Datta Meghe Institute of Medical Sciences, Wardha, India; 6 Aga Khan University, Karachi, Pakistan; 7 Columbia University School of Medicine, New York, NY USA; 8 Instituto de Nutrición de Centro América y Panamá, Guatemala City, Guatemala; 9 Kinshasa School of Public Health, Kinshasa, Democratic Republic of the Congo; 10 University of Alabama at Birmingham, Birmingham, AL USA; 11 University of Colorado Anschutz Medical Campus, Aurora, CO USA; 12 University of Virginia School of Medicine, Charlottesville, VA USA; 13 Boston University School of Public Health, Boston, MA USA; 14 Richard M. Fairbanks School of Public Health, University of Indiana, Indianapolis, IN USA; 15 University of North Carolina School of Medicine, Chapel Hill, NC USA; 16 Moi University School of Medicine, Eldoret, Kenya; 17 Sidney Kimmel Medical College—Thomas Jefferson University, Philadelphia, PA USA

**Keywords:** Risk factors, Preventive medicine

## Abstract

**Objective:**

To identify risk factors for bag and mask ventilation (BMV) and compare mortality outcomes between infants who did and did not receive BMV.

**Study design:**

Secondary analysis of data from the seven sites of the Global Network Maternal Newborn Health registry, including fresh stillbirths and live births ≥ 1500 grams from 2017 to 2023.

**Results:**

A total of 171 279 births (98.9% live births, 1.1% fresh stillbirths) were included. BMV was administered in 3.9% of cases. Maternal education, labor complications, delivery location, and prematurity were identified as major risk factors for receiving BMV. Adjusted relative risks (95% CI) for very early neonatal mortality with BMV was 26.18 (23.02–29.79), early neonatal mortality 19.08 (17.40–20.93), neonatal mortality 14.23 (13.08,15.49, mortality < 42 days 12.86 (11.84,13.97) and asphyxia-related deaths was and 2.42 (2.11, 2.77).

**Conclusion:**

These finding underscore the importance of early identification of maternal and perinatal factors associated with receiving BMV to improve neonatal outcomes.

## Introduction

Approximately one million neonates die annually in low and middle-income countries (LMICs) due to failure to initiate breathing at birth, a condition known as birth asphyxia [1]. Birth asphyxia is one of the primary causes of early neonatal mortality (death within the first seven days of life) [1, 2]. Approximately 98% of deaths related to birth asphyxia occur during the first week of life [3]. Severe birth asphyxia can lead to hypoxic ischemic encephalopathy (HIE), a leading cause of brain damage in early childhood and it is associated with developmental delays, cerebral palsy, intellectual and learning disabilities, and behavioral problems in children [4–6]. Birth asphyxia is also associated with emotional and financial burdens to families and society [7]. Infants who fail to establish spontaneous breathing at birth require bag and mask ventilation (BMV). BMV at birth is a lifesaving intervention for neonates. We reported that approximately 7.5% of infants in the Global Network (GN) Maternal Newborn Health Registry (MNHR) from three sites in India and Pakistan received BMV at birth [8]. Birth asphyxia is associated with several antepartum, intrapartum, and postpartum risk factors [9, 10]. Hence, exposure to BMV at birth is an indicator of antepartum and/or intrapartum physiological instability and is associated with an increased risk of HIE and death [9, 11].

Identification of antenatal risk factors associated with receiving BMV at birth in the GN cohort could help in preventing birth asphyxia, HIE, and reducing neonatal mortality in LMICs. The objective of the current study was to establish risk factors that are associated with receiving BMV at birth and compare outcomes of infants who received BMV to those who did not receive BMV at birth using data from the GN MNHR. We hypothesized that there are identifiable antepartum and intrapartum risk factors for receiving BMV at birth. We further hypothesized that very early and early neonatal mortality, neonatal mortality up to 28 days, infant mortality up to 42 days, and birth asphyxia as a cause of death through 28 days are higher in infants who received BMV at birth compared to those who did not receive BMV at birth. We used receipt of BMV as a surrogate marker for perinatal depression and birth asphyxia. Early identification of maternal and perinatal factors associated with receiving BMV at birth could enable risk stratification before birth and institute risk mitigation strategies.

## Materials/subject and methods

The GN MNHR is a prospective observational study that includes all pregnant women and delivered infants and their outcomes in defined geographic communities (clusters). The GN MNHR is supported by the *Eunice Kennedy Shriver* National Institute of Child Health and Human Development (NICHD). The NICHD GN is a multi-site research network consisting of partnerships between US institutions and international investigators and a Data Coordinating Center (DCC). For this study, sites in India (Belagavi in Karnataka and Nagpur in Maharashtra), Pakistan (Thatta in Sindh Province), Guatemala (Chimaltenango), Democratic Republic of the Congo (North and South Ubangi Provinces), Western Kenya, and Zambia (Kafue and Chongwe) were included. The Bangladesh site was excluded due to incomplete data set for the inclusion period. The included sites enrolled participants from 2017 to 2023 and had between 8 to 12 study clusters, which are defined geographic areas with ~300–500 annual births [10, 12].

The registry administrators (RAs) identified and screened all pregnant women in the study communities early in the pregnancy and prospectively followed them and their infants up to 42 days postpartum. Data were entered at each study site and transmitted through a secure process to the GN DCC. At enrollment, basic demographic information was recorded, and follow-up visits were conducted, one within 48 h of the delivery to obtain birth outcomes and the other at 42 days postpartum to record the health status of mother and baby, as described in detail elsewhere [10, 12]. Information on mother and baby was also collected at birth hospitalization. The study outcome data were based on medical record review, as well as interviews with birth attendants, the mother and the family. In addition to the prospective enrollment of pregnant women, several measures were taken to ensure accuracy of the stillbirth, maternal, and neonatal mortality data, including supervisory oversight of the RAs’ data collection and training and review of standardized definitions by research officers.

### Inclusion and exclusion criteria

The analysis included all infants whose mothers were screened, consented, and enrolled in the MNHR and expected to deliver between January 2017 and April 2023 and who had a fresh stillbirth or livebirth and a birth weight ≥ 1500 g. Infants were excluded from the analysis if mothers were lost to follow-up prior to delivery, died prior to delivery, medically terminated their pregnancy, had a miscarriage, or had a macerated or unknown type of stillbirth. Infants were also excluded if birth weight or receipt of BMV at birth was missing.

### Study outcomes

The outcomes of interest were receipt of BMV at birth and the occurrence of fresh stillbirth (fetal deaths occurring at ≥ 20 weeks’ gestation [or for those without gestational age available > 1500 g] with no signs of maceration), very early neonatal mortality on the day of birth (death < 1 day), early neonatal mortality (death < 7 days of life), neonatal mortality (death < 28 days of life), infant mortality < 42 days (death < 42 days of life), and birth asphyxia as a cause of death through 28 days. The GN cause of death algorithm was used to assign the cause of death [13]. When the GN sites are using “birth asphyxia” for the cause of death, it is referring to an imprecise diagnosis of HIE without cord blood gas, Apgar scores, formal neurological examination and brain MRI.

### Data analyses

Maternal and fresh stillbirths and infants’ demographics, clinical characteristics, and mode of delivery and outcomes were summarized across all sites and stratified by African (Democratic Republic of Congo, Zambia and Kenya), Latin American (Guatemala) and Asian (Nagpur, Belagavi, and Pakistan) sites. Demographics included education, age, parity, body mass index (BMI), number of antenatal care (ANC) visits, obstructed labor, hypertensive disorders, antepartum hemorrhage, multiple gestation and birth sex. Clinical characteristics included delivery location, birth attendant, delivery mode, birth weight, gestational age, and prematurity. The fresh stillbirth rate per 1000 fresh stillbirths plus live births, neonatal mortality rate per 1000 live births, infant mortality rate < 42 days per 1,000 live births, and causes of death through 28 days were also summarized across all sites and stratified by African, Latin American and Asian sites. As we excluded macerated stillbirth from the analysis, the denominator for the fresh stillbirth rate is fresh stillbirths plus live births and not all births.

Adjusted relative risks (aRRs) as well as associated 95% CIs of receiving BMV at birth (a binary outcome) were obtained by fitting a generalized linear model with Poisson distribution, log link, and robust (sandwich) standard errors to receiving BMV at birth with the following fixed effects: region (overall analysis only) and one of the demographic or clinical exposures. Additionally, the ‘relative variable importance’ for receiving BMV at birth with the exposures was calculated using a decision tree model in SAS HPSLIT with variance-based, impurity-reduction feature importance [14]. Finally, propensity scores for receiving BMV at birth were calculated for all participants. Variables included in the propensity score were selected based on clinical knowledge as well as variable importance, and included: education, age, parity, parity squared, body mass index (BMI), number of antenatal care (ANC) visits, obstructed labor, hypertensive disorders, antepartum hemorrhage, multiple gestation, birth sex, delivery location, birth attendant, delivery mode, low birth weight, and prematurity. Only 1.1% of records (1885/171279) had any missing covariate value. Given this very low fraction of incomplete data, missing covariate values were replaced with the mode for the categorical covariates and parity and the mean for BMI. The scores were obtained by fitting a penalized logistic regression model using Firth’s correction (penalized likelihood) with binomial distribution and logit link to receiving BMV at birth with all the above exposures. Refer to Supplementary Tables 1 and 2 for propensity score model estimates and odds ratios. For the analysis of the mortality outcomes, unadjusted relative risks (RRs) and aRRs of each outcome were obtained by fitting a generalized linear model with Poisson distribution and log link with the following fixed effects: receiving BMV at birth, region (overall analysis only), and the propensity score of receiving BMV at birth. All data analyses were done with SAS software v.9.4 (SAS Institute, Cary, NC, USA). All hypothesis tests were performed using a two-sided significance level of 0.05.

### Ethics approval

Each research site obtained approval from the local ethics review committees, the institutional review boards of partner U.S. universities, and the GN DCC. All pregnant women included in the MNHR provided informed consent for participation in the study.

## Results

A total of 186,356 women were expected to deliver between January 2017 and April 2023 at the seven GN sites. Most of the screened women (*n* = 185,212, 99.4%) were eligible, provided consent, delivered, and survived to delivery (Table 1). After excluding miscarriages, medical terminations of pregnancy, macerated or unknown type of stillbirth, missing birthweight, birth weight < 1500 g, and births with missing delivery data or receipt of BMV at birth, 171,279 births (84,534 from Africa [49.4%], 26,419 from Latin America [15.4%] and 60,326 from Asia [35.2%]) were included in the analysis. This included 169,439 live births (98.9%) and 1,840 fresh stillbirths (1.1%). A total of 6656 deliveries (3.9%) received BMV at birth (6,568 live births, 88 fresh stillbirths) and only 1% of deliveries received BMV at birth in Latin America compared to 2.2% in Africa and 7.5% in Asia.

**Table 1:** Consort Information Overall and Stratified by Region.

	Total	Africa	Latin America	Asia
Mothers screened for eligibility, *N*	186,356	87,373	27,656	71,327
Excluded, n (% of screened)^a^	13,837 (7.4)	2663 (3.0)	1058 (3.8)	10,116 (14.2)
Not eligible for MNHR	1 (0.0)	1 (0.0)	0 (0.0)	0 (0.0)
Did not consent to MNHR	87 (0.0)	0 (0.0)	83 (0.3)	4 (0.0)
Lost to follow up prior to delivery or has not delivered yet	974 (0.5)	726 (0.8)	125 (0.5)	123 (0.2)
Maternal death prior to delivery	82 (0.0)	42 (0.0)	6 (0.0)	34 (0.0)
Medically terminated pregnancy	2777 (1.5)	82 (0.1)	18 (0.1)	2677 (3.8)
Miscarriage	8266 (4.4)	904 (1.0)	669 (2.4)	6693 (9.4)
Macerated or unknown type of stillbirth	1650 (0.9)	908 (1.0)	157 (0.6)	585 (0.8)
No pregnancy-level exclusions				
Mothers, *n* (% of screened)	172,519 (92.6)	84,710 (97.0)	26,598 (96.2)	61,211 (85.8)
Fresh stillbirths and infants, *N*	174,139	85,736	26,740	61,663
Excluded fresh stillbirths or infants, *n* (% of those with no pregnancy-level exclusions)^a^	2860 (1.6)	1202 (1.4)	321 (1.2)	1337 (2.2)
Birth weight^b^ missing	162 (0.1)	109 (0.1)	3 (0.0)	50 (0.1)
Birth weight^b^ < 1500 g	2469 (1.4)	962 (1.1)	306 (1.1)	1201 (1.9)
Delivery date missing	1 (0.0)	1 (0.0)	0 (0.0)	0 (0.0)
Receipt of bag and mask ventilation missing	228 (0.1)	130 (0.2)	12 (0.0)	86 (0.1)
Fresh stillbirths or infants with no baby-level exclusions, *n* (% with no pregnancy-level exclusions)	171,279 (98.4)	84,534 (98.6)	26,419 (98.8)	60,326 (97.8)
Mothers in the analysis population, *n* (% of those screened)	169,919 (91.2)	83,652 (95.7)	26,297 (95.1)	59,970 (84.1)
Baby outcomes among those included in the analysis, *n* (%)	171,279	84,534	26,419	60,326
Fresh stillbirths	1840 (1.1)	1021 (1.2)	237 (0.9)	582 (1.0)
Live births	169,439 (98.9)	83,513 (98.8)	26,182 (99.1)	59,744 (99.0)

^a^Reasons for exclusions are mutually exclusive and presented hierarchically.

^b^Birth weight includes by measurement or estimation.

Maternal and fresh stillbirths and infants’ demographic and clinical characteristics, mode of delivery, and outcomes (overall and stratified by region) are depicted in Tables 2, 3. Approximately 34% of mothers in Asia have no schooling compared to 8.6% in Latin America. Maternal age < 20 years and ≥ 36 years was lowest in Asia (5.7% and 2.5%, respectively) compared to Latin America (17.3% and 9.6%) and Africa (22.4% and 7.4%). Body mass index (BMI) ≥ 25 kg/m^2^ was highest in Latin America (56.7%) and lowest in Asia (11%). For the whole cohort, approximately 40% of deliveries happened in hospitals, but only 18.9% of deliveries occurred in hospitals in Africa, compared to 58.4% and 62.2% in Latin America and Asia, respectively. Approximately 41% delivered at home/other in Latin America. Only 4.7% of deliveries in Africa were attended by physicians compared to 62.3% in Latin America and 53.6% in Asia. The C-section rate was lowest in Africa (2.7%), compared to 35.6% in Latin America and 28.8% in Asia. Obstructed/prolonged labor/failure to progress was lowest in Africa (4.1%).

**Table 2:** Maternal and Fetal/Neonatal Demographics and Clinical Characteristics, Overall and Stratified by Region.

	Total	Africa	Latin America	Asia
Mothers in analysis population	169,919	83,652	26,297	59,970
Maternal education, *n* (%)	169,877	83,627	26,295	59,955
No schooling	34,681 (20.4)	11,987 (14.3)	2266 (8.6)	20,428 (34.1)
Primary/Secondary	122,383 (72.0)	67,882 (81.2)	21,570 (82.0)	32,931 (54.9)
University +	12,813 (7.5)	3758 (4.5)	2459 (9.4)	6596 (11.0)
Maternal age (years), *n* (%)	169,907	83,640	26,297	59,970
<20	26,717 (15.7)	18,718 (22.4)	4552 (17.3)	3447 (5.7)
20–35	132,974 (78.3)	58,696 (70.2)	19,226 (73.1)	55,052 (91.8)
≥36	10,216 (6.0)	6226 (7.4)	2519 (9.6)	1471 (2.5)
Parity, *n* (%)	169,887	83,629	26,297	59,961
0	54,531 (32.1)	24,119 (28.8)	8702 (33.1)	21,710 (36.2)
1–2	68,914 (40.6)	30,424 (36.4)	11,100 (42.2)	27,390 (45.7)
≥3	46,442 (27.3)	29,086 (34.8)	6,495 (24.7)	10,861 (18.1)
BMI (kg/m^2^), *n* (%)	169,275	83,063	26,293	59,919
<18	16,449 (9.7)	3137 (3.8)	158 (0.6)	13,154 (22.0)
18–<25	114,011 (67.4)	62,640 (75.4)	11,216 (42.7)	40,155 (67.0)
≥25	38,815 (22.9)	17,286 (20.8)	14,919 (56.7)	6610 (11.0)
ANC visits, *n* (%)	169,795	83,593	26,283	59,919
0	2217 (1.3)	541 (0.6)	1244 (4.7)	432 (0.7)
1–3	55,640 (32.8)	32,744 (39.2)	8,107 (30.8)	14,789 (24.7)
≥4	111,938 (65.9)	50,308 (60.2)	16,932 (64.4)	44,698 (74.6)
Obstructed/prolonged labor/failure to progress, *n* (%)	11,989 (7.1)	3417 (4.1)	2759 (10.5)	5813 (9.7)
Hypertensive disorders of pregnancy, *n* (%)	3741 (2.2)	542 (0.6)	1217 (4.6)	1982 (3.3)
Antepartum hemorrhage, *n* (%)	871 (0.5)	424 (0.5)	52 (0.2)	395 (0.7)
Multiple gestation, *n* (%)	1823 (1.1)	1211 (1.4)	136 (0.5)	476 (0.8)
Fetuses/neonates in analysis population	171,279	84,534	26,419	60,326
Birth sex male, *n* (%)	88,110 (51.5)	43,488 (51.5)	13,487 (51.1)	31,135 (51.6)

**Table 3:** Delivery Characteristics and Outcomes, Overall and Stratified by Region.

	Total	Africa	Latin America	Asia
Mothers in analysis population	169,919	83,652	26,297	59,970
Delivery Location, *n* (%)	169,919	83,652	26,297	59,970
Hospital	68,452 (40.3)	15,787 (18.9)	15,366 (58.4)	37,299 (62.2)
Clinic/health center	74,159 (43S.6)	56,495 (67.5)	102 (0.4)	17,562 (29.3)
Home/other	27,308 (16.1)	11,370 (13.6)	10,829 (41.2)	5109 (8.5)
Delivery Attendant, *n* (%)	169,892	83,646	26,295	59,951
Physician	52,479 (30.9)	3953 (4.7)	16,394 (62.3)	32,132 (53.6)
Nurse/Midwife/Traditional Birth Attendant	110,085 (64.8)	75,198 (89.9)	9776 (37.2)	25,111 (41.9)
Lady Health Visitor/Family/Self/Other	7328 (4.3)	4495 (5.4)	125 (0.5)	2708 (4.5)
Delivery Mode, *n* (%)	169,916	83,649	26,297	59,970
Vaginal	141,020 (83.0)	81,426 (97.3)	16,924 (64.4)	42,670 (71.2)
C-Section	28,896 (17.0)	2223 (2.7)	9373 (35.6)	17,300 (28.8)
Fetuses/neonates in analysis population	171,279	84,534	26,419	60,326
Bag and mask ventilation, *n* (%)	6656 (3.9)	1884 (2.2)	255 (1.0)	4517 (7.5)
Birth weight^a^, *n* (%)	171,279	84,534	26,419	60,326
1500–2499 g	22,614 (13.2)	6210 (7.3)	4572 (17.3)	11,832 (19.6)
≥2500 g	148,665 (86.8)	78,324 (92.7)	21,847 (82.7)	48,494 (80.4)
Preterm, *n* (%)	23,839 (14.0)	11,353 (13.6)	2865 (10.8)	9621 (15.9)

^a^Birth weight includes by measurement or estimation.

**Fig. 1:**
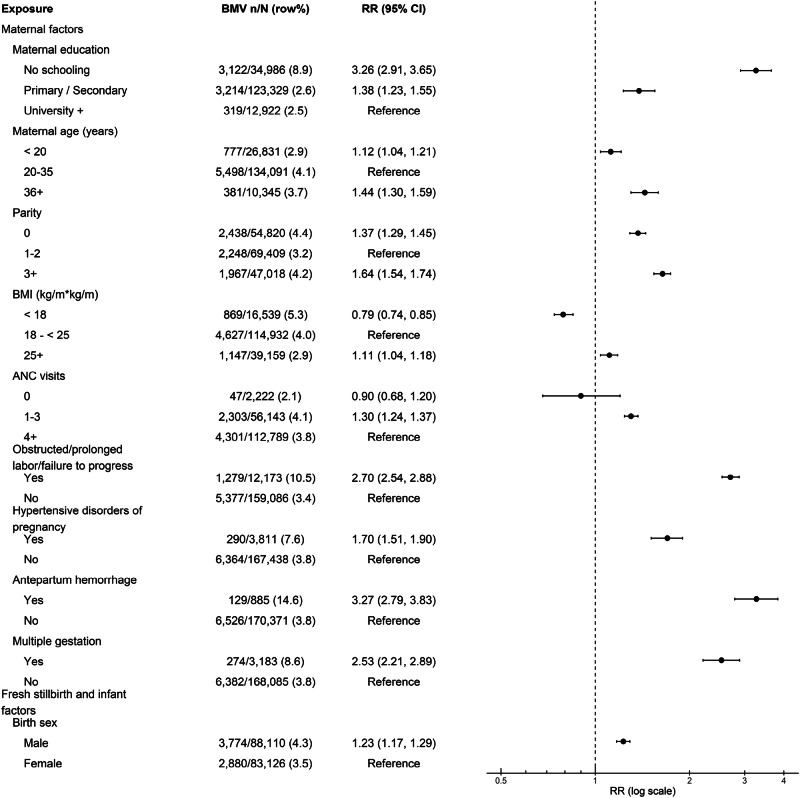
Risk factors (maternal and fetal/neonatal demographics and clinical characteristics) of receiving bag and mask ventilation. Relative risk (and associated 95% CI) of receiving BMV was obtained by fitting a generalized linear model with Poisson distribution and log link to receiving BMV with the following fixed effects: region and each exposure variable.

Maternal and fetal/neonatal demographics and clinical risk factors associated with receiving BMV at birth are depicted in Fig. 1 and Supplementary Figs. 1a–c. No schooling, obstructed/prolonged labor/failure to progress, antepartum hemorrhage and multiple gestation are associated with relative risk of > 2–3 for receiving BMV at birth. Additionally, primary/secondary education, maternal age < 20 or ≥ 36, parity of 0 or ≥ 3, BMI ≥ 25, hypertensive disorders of pregnancy, 1–3 ANC visits and male sex are associated with relative risk of > 1–2 for receiving BMV at birth. Obstructed/prolong labor/failure to progress is associated with a RR of 8.83 (8.02–9.71) for receiving BMV at birth in Africa and no schooling was associated with a RR of 4.02 (3.52–4.60) for receiving BMV at birth in Asia.

**Fig. 2:**
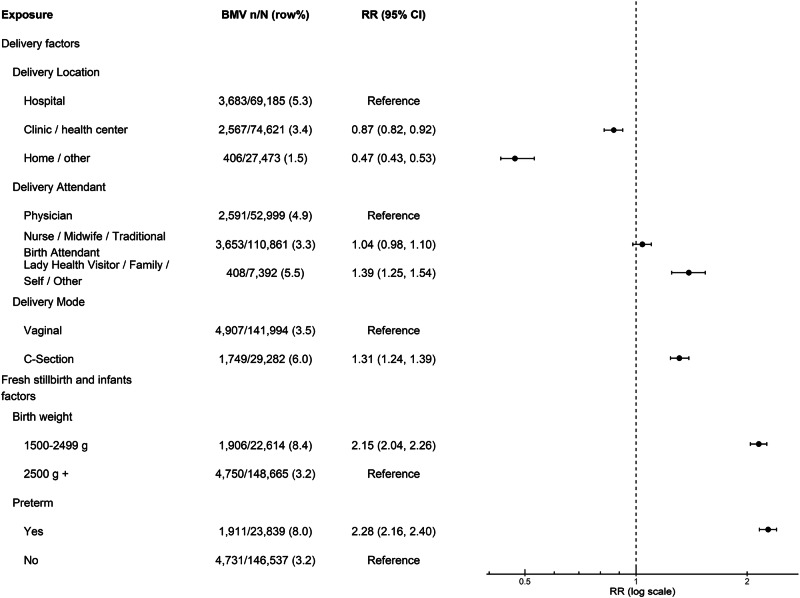
Risk factors (delivery characteristics and outcomes) of receiving bag and mask ventilation. Relative risk (and associated 95% CI) of receiving BMV was obtained by fitting a generalized linear model with Poisson distribution and log link to receiving BMV with the following fixed effects: region and each exposure variable.

Overall, clinic/health center and home/other delivery is associated with lower risk for receiving BMV, and delivery by lady health visitor/family/self/other, C-section, low birth weight and prematurity is associated with higher risk for receiving BMV at birth (Fig. 2). In Africa, delivery by nurse/midwife/traditional birth attendant and by lady health visitor/family/self/other were also both associated with lower risk for receiving BMV at birth (Supplementary Fig. 2a), and delivery at home/other and delivery by nurse/midwife/traditional birth attendant was associated with lower risk of receiving BMV at birth in Latin America (Supplementary Fig. 2b). In contrast, only home/other delivery was associated with lower risk for receiving BMV at birth and delivery by nurse/midwife/traditional birth attendant and lady health visitor/ family/self/other were associated with a higher risk for receiving BMV at birth in Asia (Supplementary Fig. 2c).

**Fig. 3:**
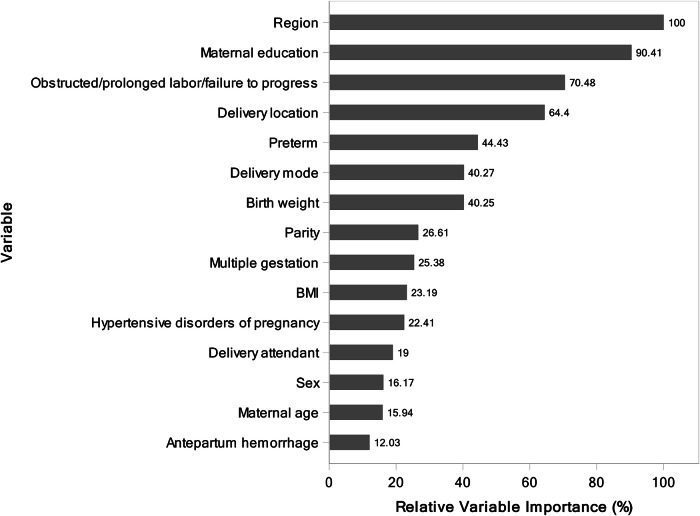
Variable importance (maternal and fetal/neonatal demographics, clinical characteristics, delivery methods and outcomes) of receiving bag and mask ventilation. Variable importance for receiving BMV calculated from SAS HPSLIP procedure which uses decision tree models with a variance-based feature importance method. The following predictors were included in the analysis: education, age, parity, body mass index (BMI), antenatal care (ANC) visits, obstructed labor, hypertensive disorders, antepartum hemorrhage, multiple gestation, birth sex, delivery location, birth attendant, delivery mode, birth weight and prematurity. Missing predictor values were replaced with the average value for the predictor. ANC is not included in the figure above, as it was not shown to be an important variable by the model.

For all regions combined, relative variable importance of receiving BMV at birth (Fig. 3) included region (100%), maternal education (90%), obstructed/prolonged labor (70%), delivery location (64%) and prematurity (44%).

Fresh stillbirth and neonatal mortality rates were 10.74/1000 fresh stillbirths plus live births and 17.52/1000 live births, respectively (Table 4). The neonatal mortality rate was highest in Asia (22.18/1000 live births) and lowest in Africa (14.47/1000 live births). Birth asphyxia, infection and prematurity were the common causes of death during the first 28 days of life. While infection was the most common cause of death in Latin America (59.9%), birth asphyxia was the most common cause in Africa (40.5%) and Asia (29.3%).

**Table 4:** Mortality outcomes, overall and stratified by region.

	Total	Africa	Latin America	Asia
Fetuses/neonates in analysis population	171,279	84,534	26,419	60,326
Fresh stillbirth, *n* (rate/1000)	1840 (10.74)	1021 (12.08)	237 (8.97)	582 (9.65)
Infants in analysis population	169,439	83,513	26,182	59,744
Neonatal mortality < 1 day, *n* (rate/1000)	1158 (6.85)	724 (8.69)	81 (3.10)	353 (5.93)
Neonatal mortality < 7 days, *n* (rate/1000)	2359 (13.96)	1063 (12.76)	282 (10.78)	1014 (17.02)
Neonatal mortality < 28 days, *n* (rate/1000)	2962 (17.52)	1205 (14.47)	436 (16.66)	1321 (22.18)
Infant mortality < 42 days, *n* (rate/1000)	3172 (18.77)	1247 (14.97)	501 (19.14)	1424 (23.91)
Cause of death, *n* (%)	2917	1172	434	1311
Congenital anomaly	190 (6.5)	40 (3.4)	37 (8.5)	113 (8.6)
Infection	868 (29.8)	281 (24.0)	260 (59.9)	327 (24.9)
Prematurity	735 (25.2)	310 (26.5)	42 (9.7)	383 (29.2)
Asphyxia	930 (31.9)	475 (40.5)	71 (16.4)	384 (29.3)
Unknown	194 (6.7)	66 (5.6)	24 (5.5)	104 (7.9)

**Fig. 4:**
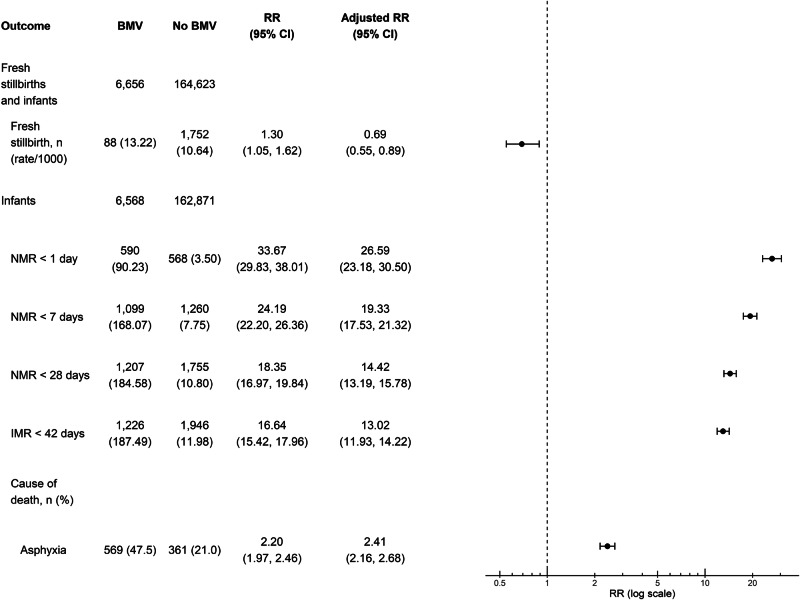
Effect of receiving bag and mask ventilation on relative risk of mortality outcomes. NMR Neonatal Mortality Rate, *n* (rate/1000 live births). IMR Infant Mortality Rate, *n* (rate/1000 live births). Relative risk (and associated 95% CI) of each mortality outcome between infants with receiving BMV and infants not receiving BMV. Estimates obtained by fitting a generalized linear model with Poisson distribution and log link to each mortality outcome with the following fixed effects: receiving BMV, region, and the propensity score of receiving BMV (adjusted RR only). The adjusted relative risk (and associated 95% CI) is provided in the forest plot.

The rate of fresh stillbirth was 13.22 per 1000 fresh stillbirths plus live births in those who received BMV at birth compared to 10.64 per 1000 fresh stillbirths plus live births in those who did not receive BMV at birth (Fig. 4) with a wide regional variation (21.76 vs. 11.86 Africa, 3.92 vs. 9.02 in Latin America, and 10.18 vs. 9.60 in Asia; Supplementary Fig. 3a–c). The relative risk of fresh stillbirth was higher in infants who received BMV at birth compared to infants who did not receive BMV at birth (RR = 1.30; 95% CI:[1.05, 1.62]). However, after adjusting for region and the propensity score of receiving BMV at birth to balance the baseline characteristics and risk factors for receiving BMV at birth between the two groups, the adjusted risk of fresh stillbirth was lower in infants who received BMV at birth than in infants who did not receive BMV at birth (aRR = 0.69; 95% CI:[0.55–0.89]). The adjusted risk for very early neonatal mortality, early neonatal mortality, neonatal mortality, and infant mortality < 42 days and birth asphyxia as a cause of death through 28 days was higher in infants who received BMV at birth than in infants who did not receive BMV at birth. Specifically, the early neonatal mortality rate ( < 7 days) was 168 per 1000 in the BMV group compared to 8 per 1000 in the no BMV group (aRR = 19.33; 95% CI:[17.53, 21.32]), neonatal mortality rate was 185 per 1000 in the BMV group compared to 11 per 1000 in the no BMV group (aRR = 14.42; 95% CI:[13.19,15.78]), and the infant mortality rate < 42 days was 187 per 1000 in the BMV group and 12 per 1,000 in the no BMV group (aRR = 13.02; 95% CI: [11.93,14.22]), and among infants who died during the first 28 days of life, 48% of infants who received BMV at birth and 21% of infants who did not receive BMV at birth died because of asphyxia ([aRR = 2.41; 95% CI:[2.16,2.68]).

## Discussion

Infants who fail to establish spontaneous breathing at birth and receive BMV at birth are at increased risk for HIE, mortality and long-term neurodevelopmental morbidities [2, 4–7, 11]. Our large population-based study involving seven GN research sites in South Asia, Africa and Latin America demonstrated that 3.9% of deliveries with birthweight ≥ 1500 g received BMV at birth, with a wide regional variation. Neonatal mortality, early infant mortality up to 42 days and birth asphyxia as a cause of death through 28 days were many folds higher in neonates who received BMV at birth. Compared to infants who did not receive BMV at birth, the adjusted risk for fresh stillbirth was lower in infants who received BMV at birth. Several maternal and neonatal characteristics and delivery methods are associated with increased risk for receiving BMV at birth which include region, maternal education, maternal age, obstructed and prolonged labor, hypertensive disorders of pregnancy, antepartum hemorrhage, multiple gestation, male sex, delivery location, delivery mode, prematurity and low birth weight.

It is estimated that about 7% of neonates in LMICs will require BMV at birth compared to 3–6% in high-income countries [15]. The lower rates of BMV in the GN centers are likely to be due to under-utilization of BMV. We excluded deliveries with birth weight < 1500 grams, which may have also contributed to a lower rate of receiving BMV at birth in our cohort. The lower rate of receiving BMV at birth in our cohort is largely due to the lower number of deliveries receiving BMV at birth in Africa and Latin America. About 7.5% of deliveries from centers in Asia received BMV at birth compared to 1% of deliveries in Latin America and 2.2% deliveries in Africa. The high proportion of home/other deliveries (41.2%) with under-utilization of BMV may have contributed to low rate of receiving BMV at birth in Latin America. Similarly, the higher number of deliveries by nurse/midwife/traditional birth attendant (89.9%) may have contributed to low rates of BMV at birth in Africa. Despite the lower rates of receiving BMV, neonatal mortality rates were lower in Africa and Latin America compared to Asia. The neonatal mortality rates at our sites in Africa (14.5/1000 live births) were lower than the neonatal mortality rates for those countries in Africa (Democratic Republic of Congo 26/1000, Kenya 20/1000 and Zambia 24/1000) (https://data.worldbank.org/indicator/SH.DYN.NMRT?skipRedirection=true&view=map). The neonatal mortality rate at sites in Asia (22.2/1000 live births) was higher than the neonatal mortality rate of 18/1000 in India but lower than 39/1000 in Pakistan. The neonatal mortality rate at our participating site in Latin America was higher (16.7/1000 live births) compared to the reported national neonatal mortality rate of 10/1000. This is despite including only deliveries with birth weight > 1500 g. Only 1% of deliveries at Latin America site received BMV at birth. This highlights the critical need for effective neonatal resuscitation training and availability of BMV at birth to improve neonatal mortality and other outcomes. We speculate that increasing use of BMV at birth in our centers in Asia, Africa and Latin America is likely to further reduce neonatal mortality rates. In Tanzania, implementation of Helping Babies Breathe (HBB) and use of BMV at birth were associated with 47% reduction in early neonatal mortality within 24 h and 24% reduction in fresh stillbirths [16]. Others have reported similar decreases in neonatal mortality and fresh stillbirth rates with implementation of HBB and use of BMV at birth [17–21]. Although HBB recommends initiation of resuscitation, including BMV for all fresh stillbirths, only 4.8% of deliveries with fresh stillbirth received BMV at birth in our cohort with a wide regional variation. Our data also supports that providing BMV at birth reduces fresh stillbirth. Providing BMV at birth to all fresh stillbirths is likely to reduce fresh stillbirth rates in LMICs.

We have identified several important risk factors for receiving BMV at birth in our cohort. Maternal education, maternal age, parity, number of antenatal visits, obstructed/prolonged labor/failure to progress, hypertensive disorders of pregnancy, antepartum hemorrhage, and multiple gestation were important maternal risk factors for receiving BMV. Other smaller retrospective chart review studies using data from single delivery hospitals have identified similar maternal risk factors for birth asphyxia [9, 22, 23]. In our cohort, delivery by c-section, low birth weight and preterm birth were also associated with increased risk for receiving BMV at birth. Similar variables have been reported to be risk factors for birth asphyxia [22]. Home delivery, an important risk factor for birth asphyxia and mortality as reported by others, was associated with lower risk for receiving BMV at birth in our cohort [22, 24]. This suggests that BMV at birth is less frequently provided in home deliveries that can potentially increase the risk for birth asphyxia and neonatal mortality. Region, maternal education, obstructed and prolonged labor, delivery location, preterm and low birth weight are important variables for receiving BMV at birth and are identified in our cohort. Results from this study indicate that several maternal risk factors that are associated with BMV at birth can be modified by improving maternal care. Identifying and understanding the risk factors associated with receiving BMV at birth is crucial in developing targeted interventions to prevent adverse outcomes, including fresh stillbirth, neonatal and infant mortality and long-term complications of HIE. Effective strategies include improving maternal education, enhancing antenatal care and visits, improving maternal health before and during pregnancy, ensuring delivery at appropriate health care facility and presence of skilled birth attendance, and providing adequate neonatal resuscitation training and resources in health care facilities.

In the current study, the receipt of BMV at birth is an independent predictor of neonatal mortality and birth asphyxia as a cause of death. This finding is not surprising as infants who are non-vigorous and do not breathe at birth require BMV. We earlier reported that approximately 25% of infants who are non-vigorous at birth develop moderate to severe HIE [11]. Birth asphyxia is one of the top three causes of neonatal mortality [1, 2]. BMV at birth is an effective intervention in neonatal resuscitation, particularly in resource-limited LMICs where access to advanced neonatal care facilities and equipment may be limited. The use of BMV at birth in LMICs has significant implications for neonatal outcomes, especially in the context of high rates of birth asphyxia. Identification of infants who are at risk for receiving BMV at birth, continued efforts to improve training for BMV at birth and consistent application, maintaining equipment and sustaining skills for BMV at birth are essential for reducing neonatal mortality and improving long-term outcomes. The knowledge of incidence and outcomes of infants who received BMV at birth will also help in planning randomized clinical trials for preventing death and long-term consequences of HIE in LMICs.

Our study has several strengths; data were analyzed from a large population-based cohort in which data are prospectively collected on maternal and infant demographic and clinical characteristics and outcomes. Common protocols and methodologies were used across the sites to document the pregnancy and neonatal outcomes, including if deliveries required BMV at birth, fresh stillbirths and neonatal and early infant mortality up to 42 days and possible causes of deaths through 28 days. Longitudinal follow up in this large cohort from early pregnancy to six weeks post-partum allowed for assessment of many maternal and neonatal characteristics and delivery methods to identify risks for receiving BMV at birth and outcomes in infants who received BMV. We calculated relative variable importance for receiving BMV at birth using a decision tree model with variance-based feature importance. We also recognize some important limitations of this study. The data were collected from limited geographical areas, and the results may not reflect overall trends for South Asia, Africa and Latin America. We did not collect data to determine the underlying cause for lower rates of BMV at birth in the African and Latin American sites. Several other important risk factors for birth asphyxia, including nuchal cord, abnormal fetal heart rate tracing and meconium-stained amniotic fluid are not collected in MNHR. Furthermore, Apgar scores, the duration of BMV at birth, and time to establish spontaneous breathing are the data variables that would have further enhanced the association and enabled better risk stratification at birth. However, these data variables are not part of the MNHR currently.

## Conclusion

In summary, our data indicate that there is a wide regional variation in deliveries receiving BMV. Early neonatal mortality, neonatal mortality, and birth asphyxia as a cause of death are higher in neonates who received BMV. There are several identifiable risk factors for receiving BMV. The results from this study show the need for better maternal education and care, creating awareness about risk factors for receiving BMV at birth to delivery providers and resuscitation team, ensuring delivery at appropriate health care facilities and presence of skilled birth attendance in high-risk deliveries, and providing adequate neonatal resuscitation training and resources in health care facilities will improve neonatal mortality, fresh stillbirth rates and reduce birth asphyxia. Our data contribute to the body of evidence suggesting the regional variation in providing BMV, risk factors associated with receiving BMV at birth and outcomes in deliveries receiving BMV at birth. The information from this study will help to develop strategies and identify measures that may help to improve use of BMV at birth and reduce infant mortality, fresh stillbirth rates, and HIE. The need for BMV at birth may also serve as an important clinical variable for neonatal risk stratification, resource reappropriation and effective neonatal surveillance for achieving better neonatal outcomes.

## Supplementary information

Supplemental Table 1

Supplemental Table 2

Supplemental Figure 1

Supplemental Figure 2

Supplemental Figure 3

## Data Availability

Individual deidentified participant data that has been captured on case report forms for pregnancy outcomes expected/obtained between January 1, 2010, and December 31, 2019, and variables that were constructed for data analysis are available indefinitely through the NICHD Data and Specimen Hub (DASH) at https://dash.nichd.nih.gov/study/20225. Data captured after January 1, 2020, will be released periodically in 3-year intervals. Other documentation includes the study protocol, case report forms, annotated case report forms, de-identification process, data dictionaries, and SAS formats. Anyone who wishes to access the data for any purpose must submit a request through NICHD DASH. All restrictions or limitations on data use will be specified in the request.

## References

[1] Newborn Health [Internet]. Available from: https://www.who.int/teams/maternal-newborn-child-adolescent-health-and-ageing/newborn-health/perinatal-asphyxia#:~:text=World%20Health%20Assembly%20%C2%BB&text=Birth%20asphyxia%2C%20defined%20as%20the,causes%20of%20early%20neonatal%20mortality.

[2] Organization WH. Maternal and Child Epidemiology Estimation Group (MCEE). Estimates for child causes of death 2000–2016. 2018.

[3] Sankar MJ, Natarajan CK, Das RR, Agarwal R, Chandrasekaran A, Paul VK. When do newborns die? A systematic review of timing of overall and cause-specific neonatal deaths in developing countries. J Perinatol. 2016;36:S1–S11. 27109087 10.1038/jp.2016.27 PMC4848744

[4] Halloran DR, McClure E, Chakraborty H, Chomba E, Wright LL, Carlo WA. Birth asphyxia survivors in a developing country. J Perinatol. 2009;29:243–9. 19037228 10.1038/jp.2008.192 PMC3807866

[5] Ahearne CE, Boylan GB, Murray DM. Short and long term prognosis in perinatal asphyxia: an update. World J Clin Pediatr. 2016;5:67–74. 26862504 10.5409/wjcp.v5.i1.67 PMC4737695

[6] Eunson P. The long-term health, social, and financial burden of hypoxic-ischaemic encephalopathy. Dev Med Child Neurol. 2015;57:48–50. 25800493 10.1111/dmcn.12727

[7] Enweronu-Laryea CC, Andoh HD, Frimpong-Barfi A, Asenso-Boadi FM. Parental costs for in-patient neonatal services for perinatal asphyxia and low birth weight in Ghana. PLoS One. 2018;13:e0204410. 30312312 10.1371/journal.pone.0204410 PMC6185862

[8] Aghai ZH, Goudar SS, Patel A, Saleem S, Dhaded SM, Kavi A, et al. Gender variations in neonatal and early infant mortality in India and Pakistan: a secondary analysis from the Global Network Maternal Newborn Health Registry. Reprod Health. 2020;17:178. 33334358 10.1186/s12978-020-01028-0 PMC7745348

[9] Abdo RA, Halil HM, Kebede BA, Anshebo AA, Gejo NG. Prevalence and contributing factors of birth asphyxia among the neonates delivered at Nigist Eleni Mohammed Memorial Teaching Hospital, Southern Ethiopia: a cross-sectional study. BMC Pregnancy Childbirth. 2019;19:536. 31888542 10.1186/s12884-019-2696-6 PMC6937931

[10] Bose CL, Bauserman M, Goldenberg RL, Goudar SS, McClure EM, Pasha O, et al. The Global Network Maternal Newborn Health Registry: a multi-national, community-based registry of pregnancy outcomes. Reprod Health. 2015;12(Suppl 2):S1. 26063166 10.1186/1742-4755-12-S2-S1 PMC4464212

[11] Girish M, Jain V, Dhokane R, Gondhali SB, Vaidya A, Aghai ZH. Umbilical cord milking for neonates who are depressed at birth: a randomized trial of feasibility. J Perinatol. 2018;38:1190–6. 29973664 10.1038/s41372-018-0161-4

[12] McClure EM, Bose CL, Garces A, Esamai F, Goudar SS, Patel A, et al. Global network for women’s and children’s health research: a system for low-resource areas to determine probable causes of stillbirth, neonatal, and maternal death. Matern Health Neonatol Perinatol. 2015;1:11. 27057328 10.1186/s40748-015-0012-7 PMC4823684

[13] Garces AL, McClure EM, Perez W, Hambidge KM, Krebs NF, Figueroa L, et al. The Global Network Neonatal Cause of Death algorithm for low-resource settings. Acta Paediatr. 2017;106:904–11. 28240381 10.1111/apa.13805 PMC5425300

[14] Leo Breiman JF, Olshen RA, Stone CJ. Classification and Regression Trees. New York 1984 1984. 368.

[15] Kamath-Rayne BD, Berkelhamer SK, Kc A, Ersdal HL, Niermeyer S. Neonatal resuscitation in global health settings: an examination of the past to prepare for the future. Pediatr Res. 2017;82:194–200. 28419084 10.1038/pr.2017.48

[16] Msemo G, Massawe A, Mmbando D, Rusibamayila N, Manji K, Kidanto HL, et al. Newborn mortality and fresh stillbirth rates in Tanzania after helping babies breathe training. Pediatrics. 2013;131:e353–60. 23339223 10.1542/peds.2012-1795

[17] Goudar SS, Somannavar MS, Clark R, Lockyer JM, Revankar AP, Fidler HM, et al. Stillbirth and newborn mortality in India after helping babies breathe training. Pediatrics. 2013;131:e344–52. 23339215 10.1542/peds.2012-2112

[18] Niermeyer S. From the neonatal resuscitation program to helping babies breathe: global impact of educational programs in neonatal resuscitation. Semin Fetal Neonatal Med. 2015;20:300–8. 26265602 10.1016/j.siny.2015.06.005

[19] Kc A, Wrammert J, Clark RB, Ewald U, Vitrakoti R, Chaudhary P, et al. Reducing perinatal mortality in nepal using helping babies breathe. Pediatrics. 2016;137. 10.1542/peds.2015-0117 27225317

[20] Versantvoort JMD, Kleinhout MY, Ockhuijsen HDL, Bloemenkamp K, de Vries WB, van den Hoogen A. Helping Babies Breathe and its effects on intrapartum-related stillbirths and neonatal mortality in low-resource settings: a systematic review. Arch Dis Child. 2020;105:127–33. 31278145 10.1136/archdischild-2018-316319

[21] Dol J, Campbell-Yeo M, Murphy GT, Aston M, McMillan D, Richardson B. The impact of the Helping Babies Survive program on neonatal outcomes and health provider skills: a systematic review. JBI Database Syst Rev Implement Rep. 2018;16:701–37. 10.11124/JBISRIR-2017-003535 29521869

[22] Aslam HM, Saleem S, Afzal R, Iqbal U, Saleem SM, Shaikh MW, et al. “Risk factors of birth asphyxia”. Ital J Pediatr. 2014;40:94. 25526846 10.1186/s13052-014-0094-2 PMC4300075

[23] Torres-Munoz J, Rojas C, Mendoza-Urbano D, Marin-Cuero D, Orobio S, Echandia C. [Risk factors associated with the development of perinatal asphyxia in neonates at the Hospital Universitario del Valle, Cali, Colombia, 2010-2011]. Biomedica. 2017;37:51–6. 28527266 10.7705/biomedica.v37i1.2844

[24] Bekele GG, Roga EY, Gonfa DN, Geda GM. Incidence and predictors of mortality among neonates admitted with birth asphyxia to neonatal intensive care unit of West Shewa Zone Public Hospitals, Central Ethiopia. BMJ Paediatr Open. 2024;8. 10.1136/bmjpo-2023-002403 PMC11002380 38580447

