# Supplementary material for: Risk factors and mortality in infants who received bag and mask ventilation at birth: a secondary analysis from the global network maternal newborn health registry
Source: J Perinatol. 2026 Jun 1;46(8):1540–9. doi: 10.1038/s41372-026-02732-8 (PMC13472852; doi:10.1038/s41372-026-02732-8)
Supplement: Supplementary file 1 [file 41372_2026_2732_MOESM1_ESM.docx]

**Supplemental Table 1: Analysis of Penalized Maximum Likelihood Estimates for Bag and Mask Ventilation Propensity Score Model**

| Parameter | DF | Estimate | Standard  Error | Wald  Chi-Square | Pr > ChiSq |
| --- | --- | --- | --- | --- | --- |
| Intercept | 1 | -3.8779 | 0.1032 | 1412.304 | <.0001 |
| Maternal Education: No Schooling | 1 | 1.7318 | 0.0642 | 727.7656 | <.0001 |
| Maternal Education: Primary/Secondary | 1 | 0.3734 | 0.0613 | 37.0898 | <.0001 |
| Maternal Age: < 20 | 1 | -0.2472 | 0.0434 | 32.3842 | <.0001 |
| Maternal Age >=36 | 1 | -0.1287 | 0.0622 | 4.2778 | 0.0386 |
| Parity | 1 | -0.1284 | 0.0152 | 71.6079 | <.0001 |
| Parity^2 | 1 | 0.0124 | 0.00173 | 51.3826 | <.0001 |
| BMI | 1 | -0.0165 | 0.00349 | 22.3443 | <.0001 |
| Antenatal care: 0 | 1 | -0.4853 | 0.1522 | 10.1659 | 0.0014 |
| Antenatal care: 1-2 | 1 | -0.0634 | 0.0293 | 4.6876 | 0.0304 |
| Obstructed labor | 1 | 1.1528 | 0.0383 | 907.4089 | <.0001 |
| Hypertensive disorders | 1 | 0.3987 | 0.0671 | 35.3129 | <.0001 |
| Antepartum hemorrhage | 1 | 0.7116 | 0.1029 | 47.8137 | <.0001 |
| Multiple birth | 1 | 0.2288 | 0.0699 | 10.728 | 0.0011 |
| Sex: Male | 1 | 0.2211 | 0.026 | 72.5578 | <.0001 |
| Delivery Location: Clinic / health Center | 1 | -0.3948 | 0.0347 | 129.2235 | <.0001 |
| Delivery Location: Home / other | 1 | -1.4383 | 0.0591 | 592.3279 | <.0001 |
| Birth Attendant: Nurse / Midwife / Traditional Birth Attendant | 1 | 0.3109 | 0.045 | 47.6758 | <.0001 |
| Birth Attendant: Lady Health Visitor/ Family / Self / Other | 1 | 0.6951 | 0.0724 | 92.212 | <.0001 |
| Mode: C-Section | 1 | 0.1915 | 0.0461 | 17.2933 | <.0001 |
| Low birth weight: 1500 - 2499 g | 1 | 0.6334 | 0.0337 | 353.5129 | <.0001 |
| Preterm | 1 | 0.5131 | 0.0332 | 238.1344 | <.0001 |

^1^ Propensity scores for bag and mask ventilation were estimated using penalized logistic regression with Firth’s correction (penalized likelihood) including the covariates which were selected based on clinical knowledge as well as variable importance.
