# Supplementary material for: Risk factors and mortality in infants who received bag and mask ventilation at birth: a secondary analysis from the global network maternal newborn health registry
Source: J Perinatol. 2026 Jun 1;46(8):1540–9. doi: 10.1038/s41372-026-02732-8 (PMC13472852; doi:10.1038/s41372-026-02732-8)
Supplement: Supplementary file 2 [file 41372_2026_2732_MOESM2_ESM.docx]

**Supplemental Table 2: Odds Ratios with Corresponding 95% Confidence Intervals for Covariates Included in the Bag and Mask Ventilation Propensity Score Model**

| Covariant | Odds Ratio | Lower 95% CI | Upper 95% CI |
| --- | --- | --- | --- |
| Maternal Education: No Schooling vs University + | 5.651 | 4.983 | 6.409 |
| Maternal Education: Primary/Secondary vs University + | 1.453 | 1.288 | 1.638 |
| Maternal Age: < 20 vs 20-35 | 0.781 | 0.717 | 0.850 |
| Maternal Age ≥ 36 vs 20-35 | 0.879 | 0.778 | 0.993 |
| BMI | 0.984 | 0.977 | 0.990 |
| Antenatal care: 0 vs ≥ 4 | 0.616 | 0.457 | 0.829 |
| Antenatal care: 1-3 vs ≥ 4 | 0.939 | 0.886 | 0.994 |
| Obstructed labor | 3.167 | 2.938 | 3.414 |
| Hypertensive disorders | 1.490 | 1.306 | 1.699 |
| Antepartum hemorrhage | 2.037 | 1.665 | 2.492 |
| Multiple birth | 1.257 | 1.096 | 1.442 |
| Sex: Male | 1.248 | 1.186 | 1.313 |
| Delivery Location: Clinic / health Center vs Hospital | 0.674 | 0.629 | 0.721 |
| Delivery Location: Home / other vs Hospital | 0.237 | 0.211 | 0.266 |
| Birth Attendant: Nurse / Midwife / Traditional Birth Attendant vs Physician | 1.365 | 1.249 | 1.491 |
| Birth Attendant: Lady Health Visitor/ Family / Self / Other vs Physician | 2.004 | 1.739 | 2.309 |
| Mode: C-Section vs Vaginal | 1.211 | 1.107 | 1.326 |
| Low birth weight (1500 - 2499 g) | 1.884 | 1.764 | 2.013 |
| Preterm | 1.670 | 1.565 | 1.783 |

^1^ Propensity scores for bag and mask ventilation were estimated using penalized logistic regression with Firth’s correction (penalized likelihood) including the covariates which were selected based on clinical knowledge as well as variable importance.
