# Supplementary material for: Risk factors and mortality in infants who received bag and mask ventilation at birth: a secondary analysis from the global network maternal newborn health registry
Source: J Perinatol. 2026 Jun 1;46(8):1540–9. doi: 10.1038/s41372-026-02732-8 (PMC13472852; doi:10.1038/s41372-026-02732-8)
Supplement: Supplementary file 3 [file 41372_2026_2732_MOESM3_ESM.docx]

**Supplemental Figure 1a: Risk Factors (Maternal and Fetal/Neonatal Demographics and Clinical Characteristics) of Receiving Bag and Mask Ventilation, Africa**

Row % = percent of fresh stillbirths and live births within each category who received BMV. Relative risk (and associated 95% CI) of receiving BMV was obtained by fitting a generalized linear model with Poisson distribution, log link, and robust (sandwich) standard errors to receiving BMV with the following fixed effects: each exposure variable. The dashed line corresponds to a RR of 1.0 depicting no association.

**Supplemental Figure 1b: Risk Factors (Maternal and Fetal/Neonatal Demographics and Clinical Characteristics) of Receiving Bag and Mask Ventilation, Latin America**

Row % = percent of fresh stillbirths and live births within each category who received BMV. Relative risk (and associated 95% CI) of receiving BMV was obtained by fitting a generalized linear model with Poisson distribution, log link, and robust (sandwich) standard errors to receiving BMV with the following fixed effects: each exposure variable. The dashed line corresponds to a RR of 1.0 depicting no association.

**Supplemental Figure 1c: Risk Factors (Maternal and Fetal/Neonatal Demographics and Clinical Characteristics) of**

**Receiving Bag and Mask Ventilation, Asia**

Row % = percent of fresh stillbirths and live births within each category who received BMV. Relative risk (and associated 95% CI) of receiving BMV

was obtained by fitting a generalized linear model with Poisson distribution, log link, and robust (sandwich) standard errors to receiving BMV with the

following fixed effects: each exposure variable. The dashed line corresponds to a RR of 1.0 depicting no association.
