# Supplementary material for: Risk factors and mortality in infants who received bag and mask ventilation at birth: a secondary analysis from the global network maternal newborn health registry
Source: J Perinatol. 2026 Jun 1;46(8):1540–9. doi: 10.1038/s41372-026-02732-8 (PMC13472852; doi:10.1038/s41372-026-02732-8)
Supplement: Supplementary file 5 [file 41372_2026_2732_MOESM5_ESM.docx]

**Supplemental Figure 3a: Effect of Receiving Bag and Mask Ventilation on Relative Risk of Mortality Outcomes, Africa**

First stillbirth= n (rate/ 1,000 fresh stillbirths + live births. NMR=Neonatal Mortality Rate, n (rate/1,000 live births). IMR=Infant Mortality Rate, n (rate/1,000 live births). Relative risk (and associated 95% CI) of each mortality outcome between infants with receiving BMV and infants not receiving BMV. Estimates obtained by fitting a generalized linear model with Poisson distribution, log link, and robust (sandwich) standard errors to each mortality outcome with the following fixed effects: receiving BMV and the propensity score of receiving BMV (adjusted RR only). The adjusted relative risk (and associated 95% CI) are provided in the forest plot. The dashed line corresponds to a RR of 1.0 depicting no association.

**Supplemental Figure 3b: Effect of Receiving Bag and Mask Ventilation on Relative Risk of Mortality Outcomes, Latin America**

First stillbirth= n (rate/ 1,000 fresh stillbirths + live births. NMR=Neonatal Mortality Rate, n (rate/1,000 live births). IMR=Infant Mortality Rate, n (rate/1,000 live births). Relative risk (and associated 95% CI) of each mortality outcome between infants with receiving BMV and infants not receiving BMV. Estimates obtained by fitting a generalized linear model with Poisson distribution, log link, and robust (sandwich) standard errors to each mortality outcome with the following fixed effects: receiving BMV and the propensity score of receiving BMV (adjusted RR only). The adjusted relative risk (and associated 95% CI) are provided in the forest plot. The dashed line corresponds to a RR of 1.0 depicting no association.

**Supplemental Figure 3c: Effect of Receiving Bag and Mask Ventilation on Relative Risk of Mortality Outcomes, Asia**

First stillbirth= n (rate/ 1,000 fresh stillbirths + live births. NMR=Neonatal Mortality Rate, n (rate/1,000 live births). IMR=Infant Mortality Rate, n (rate/1,000 live births). Relative risk (and associated 95% CI) of each mortality outcome between infants with receiving BMV and infants not receiving BMV. Estimates obtained by fitting a generalized linear model with Poisson distribution, log link, and robust (sandwich) standard errors to each mortality outcome with the following fixed effects: receiving BMV and the propensity score of receiving BMV (adjusted RR only). The adjusted relative risk (and associated 95% CI) are provided in the forest plot. The dashed line corresponds to a RR of 1.0 depicting no association.
